# Complete pathological response following chemotherapy and radiotherapy in two cases of advanced anaplastic thyroid carcinoma

**DOI:** 10.1530/ETJ-22-0111

**Published:** 2022-12-22

**Authors:** Benjamin Chevalier, Oriane Karleskind, Arnaud Jannin, Olivier Farchi, Catherine Vermaut, Alexandre Escande, Clio Baillet, Stéphanie Espiard, Marie-Christine Vantyghem, Bruno Carnaille, Emmanuelle Leteurtre, Christine Do Cao

**Affiliations:** 1 Department of Endocrinology, Diabetology and Metabolism, Lille University Hospital, Lille, France; 2 University of Lille, Lille, France; 3 Department of Pathology, Lille University Hospital, Lille, France; 4 Department of Biochemistry and Molecular Biology, Hormonology Metabolism Nutrition Oncology, Lille University Hospital, Lille, France; 5 Academic Department of Radiation Oncology, Oscar Lambret Comprehensive Cancer Center, Lille, France; 6 CRIStAL UMR CNRS 9189, University of Lille, Villeneuve-d’Ascq, France; 7 Department of Nuclear Medicine, Lille University Hospital, Lille, France; 8 Institut National de la Santé et de la Recherche Médicale (INSERM), European Genomic Institute for Diabetes (EGID), CHU Lille, Lille, France; 9 University of Lille, CNRS, Inserm, CHU Lille, UMR9020-U1277 - CANTHER - Cancer Heterogeneity Plasticity and Resistance to Therapies, Lille, France

**Keywords:** anaplastic thyroid carcinoma, sarcomatoid, complete pathological response, mismatch repair, immune microenvironment

## Abstract

**Introduction:**

Anaplastic thyroid carcinoma (ATC) is the most aggressive form of thyroid cancer with a bleak prognosis. Favorable outcomes are rare but help decipher molecular pathophysiology, investigate prognosis factors, and discover new therapeutic targets.

**Case presentation:**

Two patients were diagnosed with locally advanced nonresectable ATC, one with metastatic extension. Each patient received chemotherapy and radiotherapy, allowing thyroid surgical resection. In both cases, the pathological examination was consistent with complete response with no viable tumor cells. After follow-ups of 48 and 70 months, both patients remain disease-free. Molecular explorations on thyroid biopsies revealed microsatellite instability (MSI) and alterations on mismatch repair–gene complex, also *PTEN* and *ATM* variants in both cases. Both also presented with non-classical immune infiltrate composed of equal parts T CD4^+^ lymphocytes and macrophages.

**Conclusion:**

We report two cases of patients cured from advanced ATC and for the first time provide genetic and immunological explorations in this setting. It seems with these two cases that MSI-ATCs may indicate a better prognosis. Our study hypothesizes different responsible mechanisms including increased sensitivity to chemoradiotherapy and/or immune tumor infiltrate modulation.

**Established facts**

- Anaplastic thyroid carcinoma represents the most aggressive variant of thyroid cancer.
- Prognosis is dismal.
- Classical treatment is based on radiotherapy and chemotherapy. Surgery is rarely feasible.

**Novel insights**

- Exceptional complete pathological response can be observed in anaplastic thyroid carcinoma.
- Two cases of pathological response were associated with microsatellite instability phenotype and modulation of the immune infiltration.

## Introduction

Anaplastic thyroid carcinoma (ATC) accounts for the highest short-term mortality rate of all thyroid cancer, with almost 40–50% of thyroid cancer deaths directly related to ATC and a historical median overall survival (OS) estimated between 2 and 6 months. However, slightly better outcomes have been reported in recent years through increased knowledge of the disease’s molecular landscape and immune microenvironment, as well as the emergence of targeted therapies/immunotherapy (1, 2, 3, 4). Studying long-term cancer survivors is also a good way to improve the care of the patients. We report two ATC patient cases treated in our center over the last 10 years – including one with metastatic disease – that have demonstrated complete pathological response (i.e. without any evidence of persistent tumoral cells), confirmed by histological examination following neoadjuvant chemoradiotherapy and thyroidectomy, and no relapse during follow-up. We provide genetic and immunological explorations intended to elucidate these exceptional responses and consider new prognostic factors.

## Case presentation

### Patient 1

**Figure 1:**
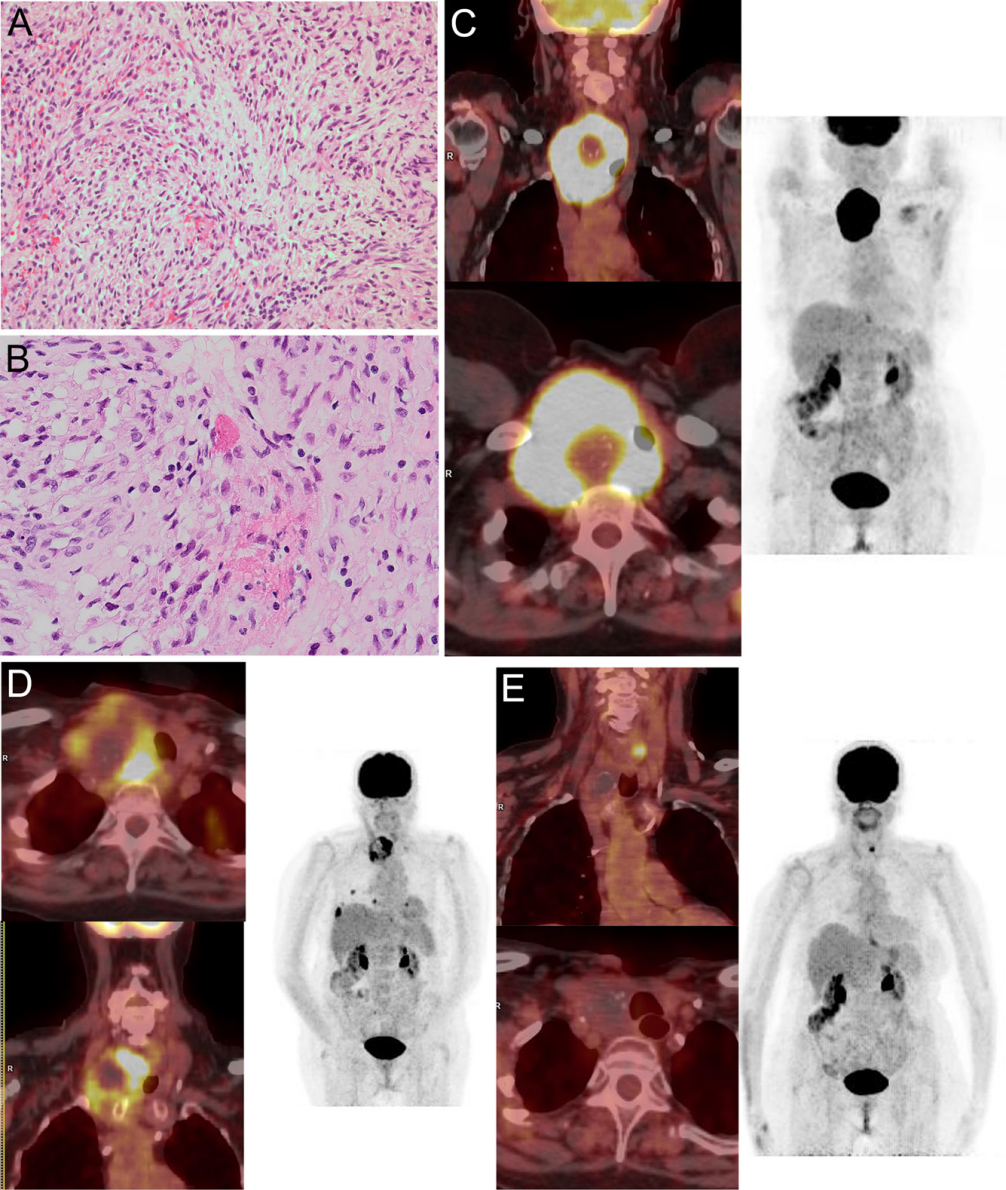
Initial presentation and evolution of patient 1. Pathological examination of thyroid biopsies (H&E stain, 100× and 400×, A-B) and ^18^FDG PET-CT results at baseline (C), after radiotherapy (D), and after chemotherapy and hypermetabolic lung nodule irradiation (E).

An 83-year-old woman without medical history was referred for a rapidly evolving 8 cm EU-TIRADS 5 thyroid mass responsible for dysphonia. Fine-needle aspiration (FNA) was assigned as Bethesda category VI, highly suggestive of undifferentiated tumor cells, and malignancy was confirmed on surgical biopsy, demonstrating a sarcomatoid ATC variant, without concurrent more well-differentiated components (Fig. 1a-b and Table 1 for immunohistochemistry (IHC) profile) (full list of antibodies detailed in supplementary data, see section on supplementary materials given at the end of this article).

**Table 1:** Patient 1 and 2 IHC profile on thyroid biopsies.

	AE1/AE3 CK	EMA	CK19	PAN CK	Caldesmon	Smooth mactinuscle	Myogenin	CS31	P40	P63
Patient 1	−	−	NA	NA	−	−	−	−	−	−
Patient 2	−	−	−	−	NA	NA	NA	NA	−	−
	**CD45**	**CD20**	**CD3**	**CD34**	**PS100**	**Desmin**				
Patient 1	NA	NA	NA	−	−	−				
Patient 2	−	−	−	NA	−	−				
					**MMR system**					
	**TTF1**	**Tg**	**PAX8**	**P53**	**Ki67**	**MLH1**	**MSH2**	**MSH6**	**PMS2**	**PD-L1**
Patient 1	−	−	−	Intermediate nuclear staining	65%	Weak	+	+	−	−
Patient 2	−	−	Weak	NA	NA	NA	−	−	+	−

−, negative stain; +, positive stain; CK, cytokeratin; EMA, epithelial membrane antigen; PAX8, paired box gene 8; PD-L1, programmed death-ligand 1; PMS2, postmeiotic segregation increased 2; MLH1, mutL homolog 1; MSH2, mutS homolog 2; MSH6, mutS homolog 6; NA, not assessed; Tg, thyroglobulin; TTF1, thyroid transcription factor-1.

Next-generation sequencing (NGS) testing (ThermoFisher Oncomine Comprehensive assay v3 panel, covering, among others, *BRAF*, *RAS*, *TP53*, *TERT* promoter, *PIK3CA* or *PTEN* genes, also *RET*-, *TRK*-, *ALK* gene fusions (full list available in supplemental data) revealed a likely pathogenic variant (Class 4 according to the ACMG guidelines for the interpretation of sequence variants) in *ATM* (c.1123 A>T, p.(Arg375*)), resulting in an early stop codon (5). A variant of unknown significance (VUS) (Class 3) was observed in *PTEN* (c.442 G>A, p.(Ala148Thr)). No *BRAF* mutation was noted. Tumor mutational burden (TMB, evaluated with tumor mutational load (TML) assay (Thermo Fisher)) was estimated to be 4.21 mutation/megabase, which is considered as low according to the 10 mutations/megabase TMB, a threshold that has been suggested to discriminate between high vs low TMB (6, 7, 8).

Molecular analysis carried out on the biopsy tissue revealed a microsatellite instability (MSI) phenotype linked to PMS2 protein expression loss. The expression of MLH1 was more questionable, with nuclear expression by tumor cells judged as weak and partial, a finding observed in patients presenting with MLH1 epigenetic inactivation (9, 10, 11). No MMR-related genes mutation was observed. Somatic *MLH1* promoter hypermethylation screening was negative (pyrosequencing on bisulfite-converted DNA (Pyromark MLH1 kit, Qiagen)). The presence of *MLH1* loss of heterozygosity (LOH) was tested: the allelic fraction for 12 SNP polymorphic markers was evaluated in tumor DNA and constitutional DNA by pyrosequencing, and on the 12 markers tested, only 1 was constitutionally heterozygous and displayed an allelic imbalance in tumor tissue, which leads to the suspicion of a LOH.

The thyroid mass showed an intense ^18^Fluorodeoxyglucose (FDG) uptake on ^18^FDG PET-CT, without evidence of extra-thyroidal metastatic dissemination (Fig. 1c). Because of a strong suspicion of gross vascular invasion on cervical CT scan, and after discussion in our Endocrine Cancer Committee, the tumor was deemed unresectable and the patient received an induction treatment with external radiotherapy (45 Gy, 30 fractions of 1.5 Gy) with persistent progressive disease of the thyroid lesion and emergence of multiple hypermetabolic lung metastases at first evaluation (Fig. 1d). The patient then received chemotherapy (carboplatin-doxorubicin monthly, six cycles) resulting in major partial response according to RECIST 1.1 criteria (−45% on cervical lesion and regression of two lung nodules) and homogeneous decrease in ^18^FDG uptake of cervical and lung lesions (12). One persistent lung metastasis that remained hypermetabolic was treated with stereotactic radiotherapy (54Gy, 3 fractions of 18Gy), resulting in a complete, local metabolic response (Fig. 1E). Treatment was fairly well tolerated, with only transient, chemotherapy-induced grade 2 alopecia and grade 1 neutropenia, also radiotherapy-induced grade 1 esophagitis and radio-epithelitis. The patient subsequently underwent total thyroidectomy and lymph node (LN) dissection. Pathological examination revealed a complete tumor response without any residual cancer cell or LN involvement. After 70 months of follow-up, the patient did not present any sign of relapse.

### Patient 2

**Figure 2:**
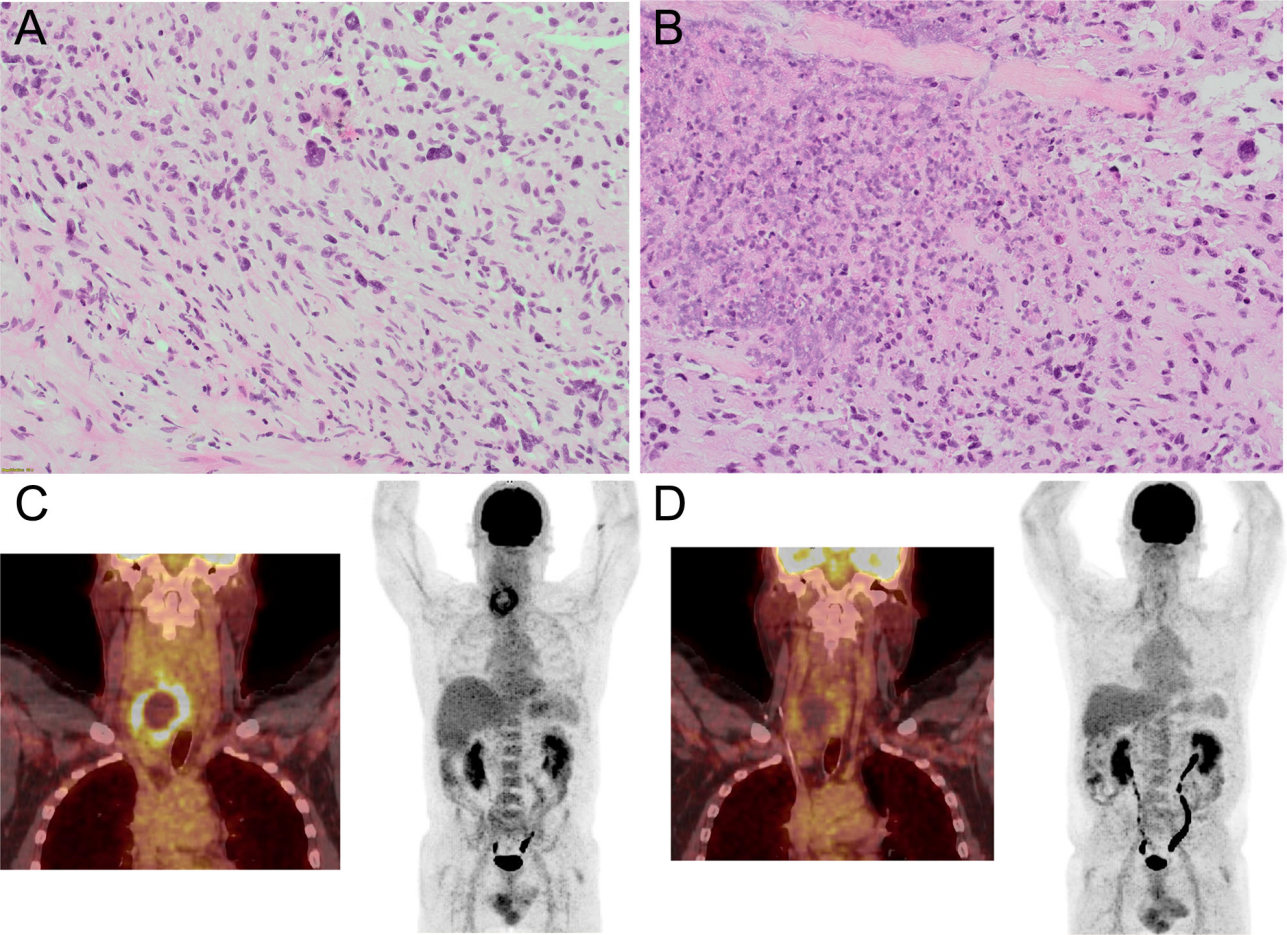
Initial presentation and evolution of patient 2. Pathological examination of thyroid biopsies (H&E stain, 200×, A-B) and ^18^FDG PET-CT results at baseline (C) and after chemo-radiotherapy (D).

A 62-year-old man had a history of a pre-existing right thyroid nodule, measuring 29 × 14 mm and described as of benign appearance with no further details on sonographic features (thyroid ultrasound (US) performed outside our center). No FNA was performed, nor any US afterwards. Three years later, he presented with a quickly evolving right cervical mass responsible for dysphonia due to right laryngeal nerve palsy and local venous compression. US revealed a right 6-cm EU-TIRADS 5 thyroid lesion, the left lobe was normal. FNA was inconclusive, and surgical biopsy revealed a sarcomatoid variant of ATC, without concurrent well-differentiated or poorly differentiated thyroid carcinoma (Fig. 2A-B, and Table 1 for IHC profile).

Molecular analysis on the biopsy showed an MSI phenotype linked to loss of MSH2 and MSH6 protein expression. Pathogenic class 5 variant was found in *TP53* (c.626_627delGA, p.(Arg209Lysfs*6)), also a class 5 *MSH2* variant (c.1857T>A, p.(Tyr619*)), a class 5 ATM variant (c.8873_8874del, p.(Phe2958*)), a class 5 *RB1* variant (c.1512_1513del, p.(Asn505Serfs*2)), a class 5 *CREBBP* variant (c.2913_2914del, p.(Arg971Serfs*20)), a likely pathogenic class 4 *PTEN* variant (c.635-3C>G, p.?), and a class 3 VUS on *KRAS* (c.183dupA, p.(Glu62Argfs*25)). No *BRAF* mutation was identified. TMB was estimated to be 13.5 mutation/megabase, considered as high.

The thyroid lesion expressed a strong avidity for ^18^FDG on PET-CT with loss of the normal esophageal wall raising the possibility of esophageal invasion, making the tumor initially unresectable, and no extra-cervical involvement was found (Fig. 2c). The patient was treated successfully with a combination of radiotherapy (56Gy, 28 fractions of 2Gy) and chemotherapy (carboplatin-paclitaxel monthly, nine cycles) resulting in a partial metabolic response with 24% decrease in the lesion size according to RECIST 1.1 criteria (Fig. 2d). Treatment adverse events were grade 1 radio-epithelitis, grade 3 esophagitis complicated with aphagia requiring transient enteral nutrition support, and chemotherapy-induced, grade 2 transient alopecia. Surgery was decided consisting of a right lobo isthmectomy to avoid the risk of contralateral recurrent laryngeal nerve injury. Pathological examination was in favor of a complete tumor response. After 48 months of follow-up, the patient did not present any sign of relapse.

### Tumor immune microenvironment exploration

**Figure 3:**
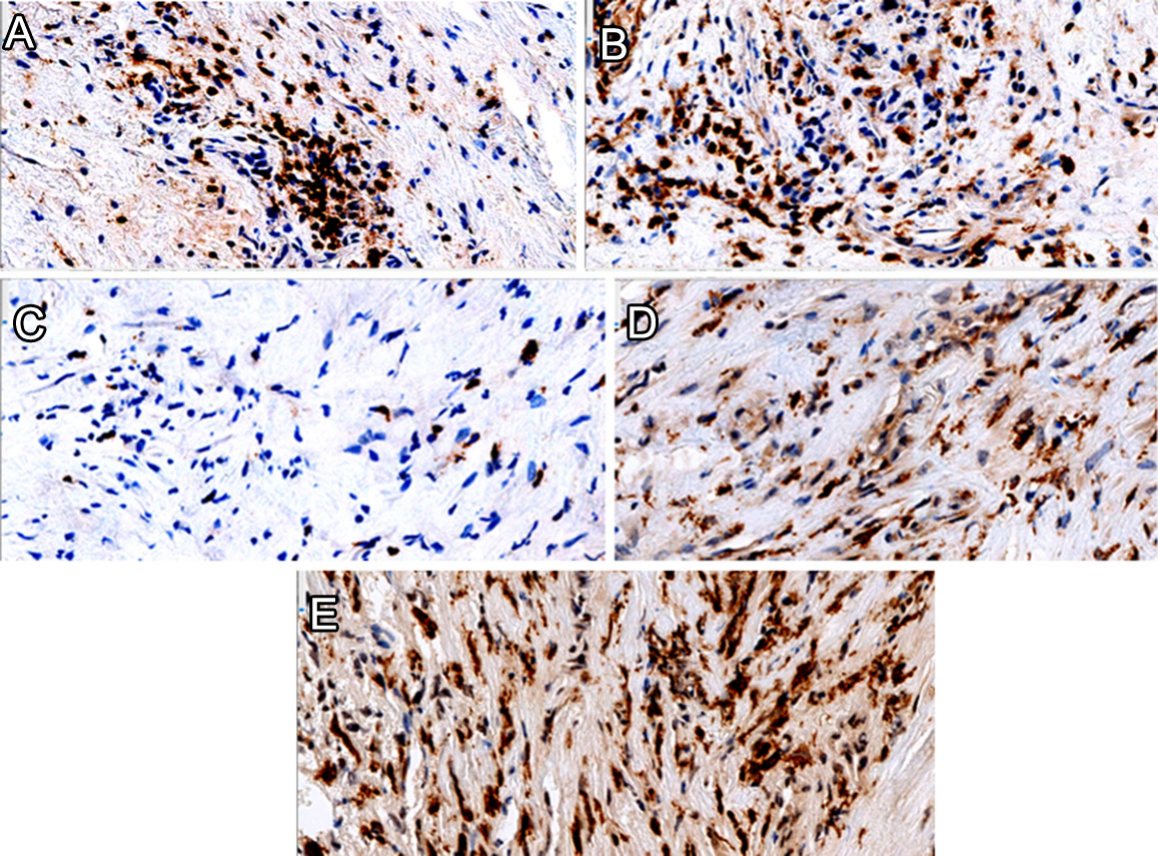
Immune infiltrate characterization. Immune infiltrate characterization with positive staining for CD3, CD4, CD8, CD68, and CD163 (3A-E, all are 400×, data from patient 2).

Immune cell characterization was similar on both patient thyroid tumor biopsies (Fig. 3A-E). IHC staining revealed that tumor microenvironment was inconspicuous and essentially composed of equal parts CD3^+^CD4^+^ T lymphocytes and CD68^+^CD163^+^ macrophages. CD8^+^T lymphocytes were proportionately less numerous than other T lymphocytes; B lymphocytes were extremely rare, and no NK cells were found. Lastly, PD-1 and PD-L1 markers were negative.

## Discussion

We report two well-characterized, exceptional cases of complete pathological tumor response in advanced stage IVb and IVc ATC. Only five similar cases of ATC patients in complete pathologic tumor response have been reported to date (Table 2), none by querying the French reference network for advanced thyroid cancer’s database (*n*  = 386, unpublished data) (13, 14, 15, 16). There was no difference between our patients and the previously reported cases regarding characteristics at diagnosis or therapeutic management, which was carried out according to standard guidelines (17, 18). Other groups have reported similar exceptional outcomes in ATC patients with complete remission, mostly after multimodal treatment effective in downsizing the tumor for surgical resection. However, we chose to not include them in our table because of the absence of complete pathological response on thyroidectomy unlike these two cases, which may reflect a difference in tumor biology (19, 20).

**Table 2:** Patient 1 and 2 characteristics and similar previously reported cases of complete pathological response.

Ref.	Sex	Age at diagnosis (years)	Thyroid tumor size (cm)	AJCC staging	RT dose	RT dose fractionation	Chemotherapy cycles (*n* = )	Nb cycles	Follow-up (months)
Patient 1	F	83	8	IVc	45 Gy thyroid 54 Gy lung nodule	1.5 Gy/fr for 30 fr 18 Gy/fr for 3 fr	Carboplatin Doxorubicin	6	70
Patient 2	M	62	6	IVb	56 Gy	2Gy/fr for 28 fr	Carboplatin Paclitaxel	9	48
Shinohara* et al*. (13)	F	53	3 × 3 × 3	IVb	69 Gy	1.8 Gy/fr for 5fr to Wilder field, then 1.2 Gy/fr BID for 50 fr to smaller field	Cisplatin Doxorubicin Etoposide Peplomycin	2	24
Abo* et al*. (14)	F	75	4.6 × 4.4 × 7.8	IVb	46 Gy	2 Gy/fr for 23 fr	Docetaxel	7	13
Zanirato Rambaldi* et al*. (15)	F	68	6	IVb	66 Gy	2.2 Gy/fr for 30 fr	Carboplatin Paclitaxel	6	32
	M	33	5	IVb	66 Gy	2.2 Gy/fr for 30 fr	Carboplatin Paclitaxel	6	27
Noguchi* et al*. (16)	M	51	3.4 × 4.1 × 3.5	IVb	40 Gy + 15 Gy IORT	Unknown	Cisplatin Doxourubicin Valproic acid	2	24

AJCC staging according to (50). BID, twice daily; CR, complete response; fr, fraction; IORT, intraoperative radiation therapy; Mx, unknown metastatic status; Nb cycles, number of cycles; RT, radiation therapy.

Data are accumulating regarding somatic molecular alterations in ATC, the most frequently mutated genes being *BRAF*, *RAS*, *TP53*, and *TERT* (21, 22, 23). Neither *TERT* nor *BRAF* mutations were observed in our two patients. A *KRAS* variant was detected in the tumor from patient 2; however, as a variant of unknown significance, pathogenicity was unclear. Moreover, the prognostic value of *RAS* mutation status has been reported with conflicting results as some authors did not find any prognostic influence whereas others recently stated that *RAS* ATC has a worse prognosis (23, 24). Patient 2 also presented *PTEN* and *TP53* variants, and patient 1 presented a *PTEN* variant too; therefore, we found in each of our two patients at least one ‘classical’ mutation frequently observed in ATC patients. Both patients potentially benefited of the absence of *TERT* promoter mutation, observed in 65–75% of ATC, consistently associated with poorer survival in large series of thyroid cancer patients, although its prognostic impact was not significant in a recent work of the MD Anderson team (21, 22, 23, 24, 25, 26, 27). The prognostic influence of *PTEN* variants, observed in both patients and classically in ATC, is unknown. The impact of the *ATM* variant observed in our two patients is otherwise difficult to hypothesize: it is usually associated with excellent radiosensitivity as observed in patient 2, but patient 1 exhibited a significantly better response to chemotherapy than to radiotherapy (28, 29).

Molecular explorations revealed a common feature in our two patients: the defect in the MMR complex involved in DNA repair (h*MLH1*, h*MSH2*, h*MSH6*, h*PMS2*), resulting in MSI. It is important to note that the IHC MMR protein profile is concordant with the presence of MSI and the *MSH2* variant harbored by patient 2, and the fact that near all reported MSI-ATC also presented with high TMB and hypermutational phenotypes (30, 31). However, no DNA mutation was found in the NGS panel for patient 1, making us suspect an epigenetic inactivation of one of the MMR genes; therefore, an *MLH1* LOH was suspected. Because MSI is classically thought to follow Knudson’s two-hit hypothesis, a second molecular hit is probable but remains unidentified. It could be due to an exonic rearrangement or an intronic variant in *MLH1*, also a variant of PMS2 is a possibility but these hypotheses could not be tested. It should be noted that no MSI related to PMS2 loss was previously reported in the context of ATC, nor has any relationship between PMS2 and TMB.

MSI is described in ~10% of ATC patients and their prognostic significance is poorly known though some authors have suggested that they could be associated with a slight increase in OS (23, 31, 32). A better prognosis of MSI-ATC could be linked to better radiosensitivity, as observed in other neoplasms such as colorectal and uterine cancer (33, 34). In contrast, there is no robust data suggesting improved chemosensitivity due to MSI.

Sarcomatoid variant of ATC was observed in our two patients but has not been associated with a better prognosis nor with cases of complete tumor response (23). In ATC, tumor stroma is composed of inflammatory elements, mainly macrophages that cooperate and interact with cancer cells and blood vessels, forming a network in which antitumor local immune control mechanisms may be impaired (35, 36, 37). Interestingly, in both tumor specimens, immune cells in the tumor microenvironment were atypically distributed since the usual predominant tumor-associated macrophages (TAMs) were equally associated with a lymphocytic infiltrate composed mainly of CD4^+^ T cells.

TAMs are classically divided into two subpopulations: M1 with anti-cancer properties and M2 that rather favor tumor development (38). With reference to ATC, a strong predominance of the M2 subpopulation contributing to tumor aggressiveness has been reported, with lower macrophage infiltration correlated with better survival performance (35, 36). This result was recently challenged by another study suggesting that ATC would rather be infiltrated by macrophages with anti-tumor activity but repressed by inhibitory molecules such as PD-L1 and CD47 overexpressed by tumor cells (37). Thus, one could hypothesize that the relatively low macrophage infiltration at diagnosis in our patients may represent a limited impairment of intratumoral immunosuppression. In contrast, we observed that CD4^+^T lymphocytes were significantly present in the tumor stroma. Lymphocytic infiltration has already been described as an indicator of ‘less poor’ prognosis in ATC (19, 37). As critical mediators of the immune response, these cells could have promoted an anti-tumor response in concert with chemoradiotherapy treatment.

Since MSI and MMR system defect has been shown to be related to CD4^+^Th1/CD8^+^T lymphocyte infiltrate, we can hypothesize that changes in intratumoral immunological landscape in our patients could potentially be linked with MSI and MMR system defect (39, 40).

The absence of overexpression of PD-1/PD-L1 observed in our two cases may also have contributed to a better prognosis since expression of these proteins, frequent in ATC, has been correlated with less favorable outcomes in ATC (41, 42, 43). However, this may be balanced by the current possibility of treating such patients with immunotherapies that have been shown to be effective such as spartalizumab (anti PD-1) (44).

Our work presents some limitations: because of the exceptional oncologic outcomes we only report here two cases, in a retrospective, monocentric way. Furthermore, our hypothesis is based on thyroid biopsies; therefore, (i) they cover a small part of the entire tumor, and heterogeneity in terms of immune cell infiltration could be a factor of bias and (ii) the amount of available tumor materials was insufficient to perform additional tests such as exome/epigenome sequencing or deeper immunophenotyping looking for macrophages/lymphocyte’s polarization.

## Conclusion

To summarize, we present the report of two cases of advanced non-*BRAF* mutated-, MSI-ATC with complete pathological response following chemotherapy and radiotherapy. Also very rare, ATC patients can be cured. This reinforces the idea that maximalist care in a referral center, including surgery, should be considered in patients who can receive these treatments, despite the severity and the aggressive nature of the disease. In a similar approach, NGS testing should be offered to all patients because of the presence of available targeted therapies in case of *BRAF* mutations (dabrafenib-trametinib) or *ALK*-, *RET*-, or *NTRK* fusions (crizotinib, ceritinib, selpercatinib, pralsetinib, larotrectinib) (45, 46, 47, 48). *CDKN2A*/*B* deletions/mutations could also potentially benefit from cell cycle inhibitors (49).

We hypothesize from these two patients that MSI increases chemoradiotherapy sensitivity in ATC by having a direct effect on tumor cells and/or indirectly by immune infiltrate modulation, increasing antitumor activity. Additional research is needed that focuses on the mechanism of response to chemoradiotherapy and the impact of the immune system on tumor cells. Indeed, deciphering patterns and pathophysiology of tumor response can help pave the way to new therapeutic strategies in this rare but particularly devastating cancer.

## Supplementary Material

Supplementary Material

## Declaration of interest

The authors have no conflict of interest to declare.

## Funding

The authors received no financial support for the research, authorship, and/or publication of this article.

## Statement of ethics

This work was conducted ethically in accordance with the World Medical Association Declaration of Helsinki. The two patients have given their written informed consent to publish their case. Information revealing subjects’ identity has been avoided. Both patients have be identified by aliases and not by their real names.

## Author contribution statement

All authors made substantial contribution to the design, analysis, and interpretation of the included data, as well as assisted with critical revisions of the writing, and approve the final version for submission for publication.
