# Supplementary material for: Complete pathological response following chemotherapy and radiotherapy in two cases of advanced anaplastic thyroid carcinoma
Source: Eur Thyroid J. 2022 Dec 22;12(1):e220111. doi: 10.1530/ETJ-22-0111 (PMC9874961; doi:10.1530/ETJ-22-0111)
Supplement: Supplementary Material [file supplementary_material.pdf]

Complete pathological response following chemotherapy and radiotherapy in two cases of advanced anaplastic thyroid carcinoma

Benjamin Chevalier, MD, Oriane Karleskind, MD, Arnaud Jannin, MD,
Olivier Farchi, MD, Catherine Vermaut, MD, Alexandre Escande, MD, Clio Baillet, MD,
Stéphanie Espiard, MD PhD, Marie-Christine Vantyghem, MD PhD, Bruno Carnaille, MD,
Emmanuelle Leteurtre, MD PhD, Christine Do Cao, MD

Supplementary Data

1. **Full list of genes covered by the NGS Oncomine assay (ThermoFisher Oncomine Comprehensive assay v3 panel).**

Hotspot genes : *AKT1, ALK, AR, ARAF, BRAF, BTK, CBL, CDK4, CHEK2, CSF1R, CTNNB1, DDR2, EGFR, ERBB2, ERB83, ERBB4, ESR1, EZH2, FGFR1, FGFR2, FGFR3, FLT3, FOXL2, GATA2, GNA11, GNAQ, GNAS, HNF1A, HRAS,IDH1, IDH2, JAK1, JAK2, JAK3, KDR, KIT, KNSTRN, KRAS, MAGOH, MAP2K1, MAP2K2, MAPK1, MAX, MED12, MET, MTOR, MYD88, NFE2L2, NRAS, PDGFRA, PIK3CA, PPP2R1A, PTPN11, RAC1, RAF1, RET, RHEB, RHOA, SF3B1, SMO, SPOP, SRC, STAT3, U2AF1, XPO1, AKT2, AKT3, AXL, CCND1, CDK6, ERCC2, FGFR4, H3F3A, HIST1H3B, MAP2K4, MDM4, MYC, MYCN, NTRK1, NTRK2, PDGFRB, PIK3CB, ROS1, SMAD4, TERT, TOP1*

Full-length genes: *ATM, BAP1, BRCA1, BRCA2, CDKN2A, FBXW7, MSH2, NF1, NF2, NOTCH1, PIK3R1, PTCH1, PTEN, RB1, SMARCB1, STK11, TP53, TSC1, TSC2, ARID1A, ATR, ATRX, CDK12, CDKN1B, CDKN2B, CHEK1, CREBBP, FANCA, FANCD2, FANCI, MLH1, MRE11A, MSH6, NBN, NOTCH2, NOTCH3, PALB2, PMS2, POLE, RAD50, RAD51, RAD51B, RAD51C, RAD51D, RNF43, SETD2, SLX4, SMARCA4*

Copy number genes: *AKT1, AR, CCND1, CCNE1, CDK4, CDK6, EGFR, ERBB2, FGFR1, FGFR2, FGFR3, FGFR4, FLT3, IGF1R, KIT, KRAS, MDM2, MDM4, MET, MYC, MYCL, MYCN, PDGFRA, PIK3CA, PPARG, TERT, AKT2, AKT3, ALK, AXL, BRAF, CCND2, CCND3, CDK2, CDKN2A, CDKN2B, ESR1, FGF19, FGF3, NTRK1, NTRK2, NTRK3, PDGFRB, PIK3CB, RICTOR, TSC1, TSC2*

Gene fusions (inter- and intragenic): *ALK, AXL, BRAF, EGFR, ERBB2, ERG, ETV1, ETV4, ETV5, FGFR1, FGFR2, FGFR3, NTRK1, NTRK3, PDGFRA, PPARG, RAF1, RET, ROS1, AKT2, AR, BRCA1, BRCA2, CDKN2A, ERB84, ESR1, FGR, FLT3, JAK2, KRAS, MDM4, MET, MYB, MYBL1, NF1, NOTCH1, NOTCH4, NRG1, NTRK2, NUTM1, PDGFRB, PIK3CA, PRKACA, PRKACB, PTEN, RAD51B, RB1, RELA, RSPO2, RSPO3, TERT*

2. Pathology procedure and antibodies used for the study of tumor immune microenvironment.

The formalin-fixed paraffin-embedded (FFPE) and haematoxylin, eosin and safran-stained histological sections of thyroid biopsies and thyroidectomy were evaluated by expert thyroid pathologist. Immunohistochemistry (IHC) was performed on 4 µm thick sections of FFPE thyroid biopsy tissue with classical antibodies used in thyroid pathology, including anti TTF1, thyroglobulin, PAX8, chromogranin A, CD20, CD3, CD45, PS100, caldesmon, desmin, smooth muscle actin, myogenin, pancytokeratin, cytokeratin 19, cytokeratin AE1/AE3, EMA, P53, P63, P40 and Ki-67.

Additional IHC analysis were performed with the following markers in order to analyze the intratumoral immune infiltrate: CD20 (L26 clone, Bio SB) and CD79α (JCB117 clone, Dako) targeting B lymphocytes and plasmocytes, CD3 (LN10 clone, Leica), CD4 (SP35 clone, Ventana Roche) and CD8 (C8/144B clone, Dako) targeting T lymphocytes, CD56 (CD564

clone, Leica) targeting Natural Killer cells, CD68 (KP1 clone, Ventana Roche) and CD163 (K20-T clone, Biotech) expressed by macrophages, the co-inhibitory molecules PD-1 (NAT105 clone, Ventana Roche) and PD-L1 (22C3 clone, Dako), finally MLH1, PMS2, MSH2 and MSH6 (G168-728, A16-4, G219-1129, 44 clones respectively, all from Ventana Roche), in order to evaluate the expression of MMR proteins.
